# What is the role of visual skills in learning to read?

**DOI:** 10.3389/fpsyg.2014.00776

**Published:** 2014-07-24

**Authors:** Yanling Zhou, Catherine McBride-Chang, Natalie Wong

**Affiliations:** ^1^Department of Early Childhood Education, The Hong Kong Institute of EducationHong Kong; ^2^Developmental Centre, Department of Psychology, The Chinese University of Hong KongHong Kong

**Keywords:** reading, visual skill, Chinese, orthography, visual processing

Although the issue of visual skills in relation to word reading has not been central to recent explorations of reading development, all visual word reading involves visual skill. Children constantly face tasks of differentiating visually similar letters or words. For example, distinguishing “b” from “d,” “a” from “e,” or “book” from “boot” all require visual differentiation. Children's orthographic knowledge and letter knowledge are causal factors in subsequent reading development in English (e.g., Badian, 1994; Lonigan et al., 2000). At a pure visual skill level, some researchers (e.g., Franceschini et al., 2012) suggest that core visual processing skills such as visual spatial attention in preschoolers could be a causal factor in subsequent reading acquisition. In addition, some alphabetic readers with dyslexia may have visual processing deficits (e.g., Valdois et al., 2004; Van der Leij et al., 2013). Following this hypothesis, Franceschini et al. (2013) showed that action video games that strengthened children's visual attention also improved their reading speed in Italian without sacrificing reading accuracy, similar to previous interventional research training facilitating visuospatial attention skills in Italian children with dyslexia (Facoetti et al., 2003). However, orthographic depth mediates the role of visual attention in reading (Bavelier et al., 2013; Richlan, 2014). English is a more opaque orthography than Italian, and Chinese is even more opaque than English.

Both eye movement and neuroimaging studies have demonstrated that reading Chinese affects visual processing differently than does reading alphabetic orthographies (e.g., Inhoff and Liu, 1998; Perfetti et al., 2010; Szwed et al., 2014). Inhoff and Liu (1998) found that Chinese readers used comparatively smaller visual perceptual spans than English readers. Szwed et al. (2014) found that readers of Chinese showed strong activations in intermediate visual areas of the occipital cortex; these were absent in French readers. Researchers have attributed these characteristics to perceptual learning resulting from learning to read Chinese characters (Rayner, 1998; Perfetti et al., 2010; Szwed et al., 2014).

Indeed, the role of visual skills for early reading development may be stronger for reading Chinese than reading English. Pure visual skills are sometimes relatively strong correlates of Chinese children's reading (e.g., Huang and Hanley, 1994, 1995; Ho and Bryant, 1997; Siok and Fletcher, 2001; Mcbride-Chang et al., 2005; Luo et al., 2013). Such visual tasks likely tap at least three visual skills that may be required for Chinese character reading. First, a focus on visual form constancy (e.g., is this square the same size as the one embedded in other designs on the previous page?) likely has some analogies with the fact that radicals within Chinese might appear as larger or smaller or even reversed in appearance across characters. Second, a focus on visual spatial skills, i.e., identifying the same form when it is in a different direction or placed differently, might be useful when Chinese character identification requires children to reduce a compact character into component radicals. Third, visual memory may be useful in learning to read Chinese in at least two ways, namely, making associations between characters and sounds, many of which are arbitrary, sometimes referred to as visual verbal paired associate learning, and helping children to build a mental memory of how different radical parts are located within a character.

What is the evidence that learning to read Chinese trains one's visual skills? A cross-cultural study (McBride-Chang et al., 2011b) found that Chinese and Korean kindergartners performed significantly better than Israeli and Spanish children on a task of visual spatial relationships, the only visual task tested across all four cultures. Korean kindergartners tend to learn to read Korean syllables holistically initially, similar to how Chinese characters are taught. A superior performance on visual skills was also found by Demetriou et al. (2005) for older Chinese as compared to Greek children. In addition, Huang and Hanley (1994) found that both Taiwanese and Hong Kong children showed a clear advantage on the visual form discrimination task as compared to their British peers.

Interestingly, the Chinese written system has two versions, the simplified and the traditional. The simplified script, which has fewer visual features to distinguish one character from another, may make more visual demands than does the traditional version. In one study of those learning to read traditional (in Hong Kong) as compared to simplified (in Mainland China) script, those learning the simplified script outperformed those learning the traditional one on three visual tasks, namely visual discrimination, visual spatial relationships and visual closure tasks (Mcbride-Chang et al., 2005), even across time. Peng et al. (2010) found that electrophysiological response potentials (ERPs) in the brains of those expert readers who saw characters with one stroke either added or subtracted in a few milliseconds showed the same basic pattern: The brains of simplified script readers appeared to register the alterations early in processing; those of traditional script readers did not.

Importantly, most studies on the topic of visual skills and word reading were carried out at a single point in time, rather than in a longitudinal study, and there are few experimental studies on this topic. Thus, the issue of causality is not clear (e.g., McBride-Chang et al., 2011b; Yang et al., 2013). However, there is probably a bidirectional association between pure visual skills and learning to read in the early years (e.g., McBride-Chang et al., 2011b). Future directions in this area should focus on at least three aspects of research in order to gain a better understanding of the causal associations between literacy and visual skills. First, more research should explore how and which visual skills may promote word reading in the early grades. Second, researchers can consider more broadly how learning to read particular orthographies might facilitate given visual skills. Third, an exploration of pure visual skills and word reading could be expanded further to visual-motor skills and writing.

The issue of how visual skills facilitate word reading is important to explore in a variety of orthographies. For example, Nag (2007, 2011) has presented a model of orthographically contained vs. extensive orthographies. At the most extensive level is Chinese, with thousands of possible characters. At the most contained level are alphabetic orthographies with sets of around 24 to 30 letters each. In the middle, she placed Bengali, Hindi, and Kannada, each with 400+ possible symbols. Collectively, these are referred to as the ashkara languages. As with Chinese, small changes in visual symbols in ashkara scripts signal potentially large changes in phonological or meaning representations. For example, native readers of Arabic are slower to process it than a second language of Hebrew because of differences in visual complexity (Ibrahim et al., 2002; Abdelhadi et al., 2011). Even in simple alphabetic orthographies, young children might memorize words based on particular visual features (e.g., M has two humps; the word “bed” in English actually looks like a bed). Therefore, it is important for researchers to continue to explore whether and which visual analysis skills might explain early literacy in diverse orthographies. Perhaps more varied visual skills would best explain performances in ashkara languages and Chinese given their visual complexities. For this research question, the focus is on individual differences within a group learning to read in a single orthography.

The second question focuses more on group differences across orthographies: Do orthographies facilitate visual flexibility and analysis in different ways? Perhaps not only are the visual characteristics of the orthography important, but the style of teaching and learning the orthography is additionally important for facilitating visual skills. For example, although Korean Hangul is ultimately a relatively simple alphasyllabary, it is taught initially to children more in the form of syllables with different components, such that children have many different configurations to learn. Perhaps children's visual memorization loads would be reduced if they were taught the basic phonological rules of Korean first, but this is not the way in which they are instructed. Future research should consider both the dimensions of visual demands of the orthography and also teaching approaches. For example, young children are often taught to read Thai with spaces in between words indicated before they are gradually coaxed to read Thai as it is written for adults—without spaces between words. Conceivably, those learning to read Thai with and without spaces might show different visual patterns of discrimination early on. Another area for research on this topic would be to compare children learning to read orthographies that differ on the dimension of contained vs. expansive as defined by Nag, at least in three aspects, i.e., alphabetic, ashkara, and Chinese, across the early years of literacy development (perhaps ages 4–7 years) to determine whether those learning an alphabet are least sophisticated in visual skill, those learning an ashkara in the middle, and Chinese learners the best. Although McBride-Chang et al. (2011b) compared 5-year-olds learning Chinese, Korean, and alphabets, this study could be expanded to include ashkara learners and to examine the developmental trend with age across several dimensions of basic visual skill.

A third issue to consider makes an analogy from word reading and visual recognition, considered above, to word writing and visual copying. We have recently established that children who tend to write Chinese characters better tend also to show better abilities to copy 2-dimensional unfamiliar forms (e.g., Wang et al., 2013). These forms were foreign scripts that were coded by those unfamiliar with each (e.g., a Chinese research assistant evaluated children's writing of Hebrew based purely on the visual representation of the writing perceived). Unfamiliar scripts were selected to ensure that the 2-dimensional writing was equally unfamiliar to all children. (In contrast, copying of geometric shapes or one's own script would be potentially problematic because those who are academically more skilled often tend to write all familiar stimuli better than those who are less so). Tan et al. (2005) have suggested that copying skills are important for learning to write/spell Chinese. However, there is very little research that has examined this question for orthographies other than Chinese. Vellutino et al. (1975) found that the copying of Hebrew did not distinguish dyslexic from non-dyslexic children who were native English speakers unfamiliar with Hebrew. In contrast, such copying skills did distinguish those with and without dyslexia in Chinese (McBride-Chang et al., 2011a). However, few, if any researchers, have gone further with this research, examining to what extent the ability to copy unfamiliar materials is associated with the ability to write words in a native orthography. Given a proposed first stage of word reading as primarily visual (Ehri, 2013), a first stage of word writing might be, correspondingly, associated with visual-motor skills that can be measured using pure two-dimensional patterns. Perhaps such copying abilities are particularly linked to learning to write in Chinese or in ashkara. However, it is important to examine these abilities independently within, as well as across, orthographies. While it may be the case that copying of unfamiliar stimuli explains subsequent spelling skills for Hindi, Chinese, Korean, or even Dutch children within a group, for example, it is also interesting to consider whether learning to read the most expansive orthography of Chinese facilitates superior visuo-motor skills more generally (as compared to those learning to read and write Dutch, for example). More research on the interface between literacy skills within and across orthographies and visual and visuo-motor skills, can potentially yield new directions that are theoretically interesting and possibly practically important.

To conclude, the role of visual skills in learning to read is apparently complex, and our understanding of this role depends upon the extent to which we look within as compared to between orthographies. The types of visual skills, the types of orthographies, and the teaching methods for literacy instruction all influence this association. Moreover, the association between visual skills and literacy development is likely bidirectional. This association can be expanded to focus on visuo-motor skills and writing. These issues cross-culturally represent some new avenues for future research.

## Conflict of interest statement

The authors declare that the research was conducted in the absence of any commercial or financial relationships that could be construed as a potential conflict of interest.
